# Novel Approaches and Challenges of Discovery of Exosite Modulators of a Disintegrin and Metalloprotease 10

**DOI:** 10.3389/fmolb.2020.00075

**Published:** 2020-05-06

**Authors:** Dmitriy Minond

**Affiliations:** ^1^Rumbaugh-Goodwin Institute for Cancer Research, Nova Southeastern University, Fort Lauderdale, FL, United States; ^2^Dr. Kiran C. Patel College of Allopathic Medicine, Nova Southeastern University, Fort Lauderdale, FL, United States

**Keywords:** ADAM10, drug discovery, exosite, inhibitors, glycosylation

## Abstract

A disintegrin and metaproteinase 10 is an important target for multiple therapeutic areas, however, despite drug discovery efforts by both industry and academia no compounds have reached the clinic so far. The lack of enzyme and substrate selectivity of developmental drugs is believed to be a main obstacle to the success. In this review, we will focus on novel approaches and associated challenges in discovery of ADAM10 selective modulators that can overcome shortcomings of previous generations of compounds and be translated into the clinic.

## Introduction

**FIGURE 1:**
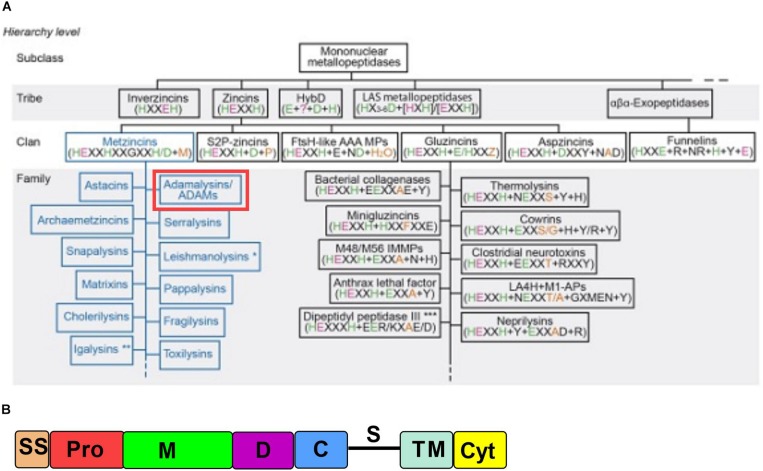
ADAM10 is a part of a large class of proteolytic enzymes. **(A)** ADAM family’s place in metalloprotease hierarchy. Reproduced with permission from Cerda-Costa and Gomis-Ruth (2014). **(B)** ADAM10 domain organization. SS, signal sequence; Pro, prodomain; M, metalloproteinase; D, disintegrin; C, cysteine-rich; S, stalk region; TM, transmembrane; Cyt, cytoplasmic tail. (Adapted from Seegar et al., 2017).

A disintegrin and metaproteinase 10 (ADAM10) is member of a large group of human and non-human zinc-dependent enzymes (reviewed in Cerda-Costa and Gomis-Ruth, 2014). Structurally it belongs to the adamalysin family (Figure 1A, ADAM and ADAMTS enzymes). ADAM10 is a cell surface enzyme that sheds a wide variety of cell surface proteins (Dreymueller et al., 2015; Kuhn et al., 2016; Camodeca et al., 2019; Scharfenberg et al., 2019) with importance in the progression of cancer, inflammation and immune response, suggesting that ADAM10 can be an important target for therapy.

ADAM10 is comprised of several domains, namely signal sequence, prodomain, metalloproteinase domain, disintegrin domain, cysteine-rich domain, stalk region, transmembrane domain, and cytoplasmic tail (Figure 1B), which are common for adamalysins (Takeda, 2009, 2016). ADAM10’s most closely related adamalysin is ADAM17 with which it shares overall 24% amino acid sequence homology (as analyzed by Clustal Omega alignment tool). Despite low sequence homology ADAM10 and ADAM17 have a broadly overlapping and ever growing substrate repertoire, possibly due to the lack of well-defined cleavage site primary sequence specificity (Caescu et al., 2009).

Functions of ADAM10 in any particular disease or normal physiological scenario are defined by the substrates that it cleaves; however, it is not well-known if ADAM10 and ADAM17 cleave the same substrates in the same setting. Therefore, inhibitors selective for ADAM10 can help differentiate its role in various scenarios.

Ability to cleave multiple substrates further complicates studies of ADAM10’s role and, therefore, its validation as a target for any particular disease. ADAM10 cleaves receptors and receptor ligands such as cytokines, chemokines, cell adhesion molecules to name a few (Caescu et al., 2009; Pruessmeyer and Ludwig, 2009; Dreymueller et al., 2015; Saftig and Lichtenthaler, 2015; Moss and Minond, 2017; Wetzel et al., 2017). An ADAM10 selective inhibitor that binds to a zinc of an active site will prevent proteolysis of all ADAM10 substrates. Given that ADAM10 substrates can counteract each other’s biological effect (e.g., pro- and anti-inflammatory cytokines), a substrate-specific inhibitor of ADAM10 can be useful.

This notion lead to the deeper exploration of regulatory mechanisms governing recognition and interaction between ADAM10 and ADAM17 and their substrates by several groups, including ours. These studies led to the realization that ADAM10 and ADAM17 may have multiple levels or ways of regulation of substrate recognition and processing that are outside of their active sites. Among the regulatory mechanisms known so far are trafficking of ADAMs (Lorenzen et al., 2016; Matthews et al., 2017; Seipold et al., 2018), interactions with other proteins (Koo et al., 2020), cellular membrane re-arrangement (Reiss and Bhakdi, 2017), ADAMs non-catalytic domains (Willems et al., 2010; Tape et al., 2011; Stawikowska et al., 2013; Seegar et al., 2017), topology of ADAM substrates (Stawikowska et al., 2013), enzyme (Chavaroche et al., 2014), and substrate glycosylation (Minond et al., 2012). As demonstrated by several groups these regulatory mechanisms can be targeted for a modulator discovery (Tape et al., 2011; Minond et al., 2012; Madoux et al., 2016; Seegar et al., 2017).

There has been a significant effort dedicated to the discovery of modulators of ADAM10 activity for multiple indications such as rhematoid arthritis (RA) (Moss et al., 2008a), cancer (Moss et al., 2008b; Crawford et al., 2009; Saha et al., 2019), immune and neurodegenerative disorders (Wetzel et al., 2017). It is important to note, that for some indications (e.g., Alzheimer’s disease) molecules that induce or potentiate ADAM10 activity are thought to be needed, whereas for the majority of other indications (e.g., cancer, inflammation) the inhibitors of activity are sought after.

**FIGURE 2:**
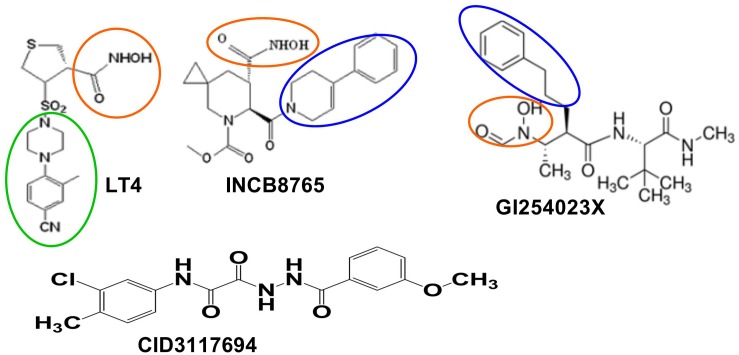
ADAM10 selective inhibitors. Zinc-binding moieties are in red circles. Modeling suggests bulky aromatic group (in the green circle) of LT4 interacts with S1’ exosite of ADAM10. Bulky aromatic groups of INCB8765 and GI254023X potentially interacting with ADAM10 S1’ site are in the blue circles. CID3117694 does not have apparent zinc-binding groups.

There are several selective inhibitors of ADAM10 that are available to the researchers, including LT4 (ADAM10 IC_50_ = 40 nM, ADAM17 IC_50_ = 1500 nM; Zocchi et al., 2016), INCB8765 (Incyte Corporation, ADAM10 IC_50_ = 97 nM, ADAM17 IC_50_ = 2045 nM; Zhou et al., 2006), GI 254023X (Glaxo, ADAM10 IC_50_ = 5.3 nM, ADAM17 IC_50_ = 541 nM; Ludwig et al., 2005), and ADAM10 prodomain (Biozyme Inc., ADAM10 IC_50_ = 48 nM, ADAM17 IC_50_ > 10 μM; Moss et al., 2007). LT4, INCB8765 and GI254023X are small molecules containing hydroxamate moieties and, therefore, likely to inhibit ADAM10 *via* a Zn-binding mechanism (Yiotakis and Dive, 2008) (Figure 2). ADAM10 prodomain is a competitive inhibitor of ADAM10, but it is unknown whether it binds the active site Zn. While Zn-binding inhibitors can exhibit a degree of selectivity between closely related ADAM family members, they ultimately cannot selectively inhibit shedding of substrates. There is evidence that toxicity has been caused by off-target side effects (Dekkers et al., 1999; Newton et al., 2001; Moss and Bartsch, 2004) due to a Zn-binding mechanism of inhibition which results in broad spectrum inhibition of multiple Zn metalloproteases. Additionally, ADAM10 has been shown to cleave > 70 cell surface proteins; therefore, indiscriminate inhibition of shedding of these proteins can affect multiple biological processes (reviewed in Dreymueller et al., 2015; Wetzel et al., 2017).

As shown by global knockout studies, ADAM10 (Hartmann et al., 2002) is vital for development, homeostasis and repair, which makes global inhibition of all functions of this enzyme non-feasible as a therapeutic approach. However, tissue-specific partial knockout studies of ADAM10 (Chalaris et al., 2010) demonstrated the lack of overall toxicity suggesting that local pharmacological partial inhibition of ADAM10 can be used.

Our group has discovered a new class of selective ADAM10 inhibitors that act *via* a non-Zn-binding mechanism (Madoux et al., 2016) and potentially bind outside of an active site (Figure 2). This non-Zn-binding mechanism of inhibition proved to be the key for ensuring selectivity of these molecules toward other Zn metalloproteinases. Additionally, the lead compound CID 3117694 from this new chemotype exhibits a unique *substrate selectivity* profile (Madoux et al., 2016) not observed with Zn-binding inhibitors of ADAM10, which should help avoid the off-target side effects described for Zn-binding inhibitors of ADAM10. For example, inhibition of shedding of amyloid precursor protein (APP) by ADAM10 (Fahrenholz, 2007) could lead to amyloid plaque formation in CNS. Additionally, many of Zn-binding inhibitors of metalloproteinases caused a dose-limiting toxicity known as Musculo-Skeletal Syndrome (MSS) (Overall and Lopez-Otin, 2002).

Search of PubChem database for biological activity of CID 3117694 revealed that it was inactive in 524 bioassays and active only against 3 targets with ADAM10 being a top target (PubChem AID 743338). Second target was hERG – CID 3117694 protected hERG from pro-arrhythmic agents (PubChem AID 1511, no EC_50_ value reported). Third target was DNA polymerase β (PubChem AID 485314) where CID3117694 exhibited IC_50_ value of 79 μM. It was inactive against adrenergic (ADRB2), muscarinic (CHRM1) and opioid receptors (OPRK1, OPRM1, and OPRD1) which are used for drug candidate safety screens (Bowes et al., 2012). These data suggest that CID 3117694 is a non-promiscuous compound which should translate into low off-target *in vivo* toxicity. This also suggests that inhibition of ADAM10 *via* a non-Zn-binding mechanism could be an effective strategy for therapy with fewer side effects due to enzyme and substrate selectivity superior to Zn-binding inhibitors.

In the review presented herein we will discuss approaches and challenges of rational design and discovery of enzyme- and substrate-selective modulators of ADAM10.

## Article

As mentioned above, there are multiple considerations and challenges in the development of small molecule therapy targeting ADAM10. Firstly, ADAM10 modulators need to be able to avoid affecting ADAM17 (and other metzincins) with which they share multiple common substrates (Caescu et al., 2009). Additionally, since ADAM10 sheds multiple substrates, depending on the particular therapeutic indication, its modulators might need to be substrate-selective. ADAM17 selective inhibitors of ADAM10 have been reported (Figure 2 and Table 1). All ADAM10 substrates interact with a catalytic zinc atom of an ADAM10’s active site, therefore, modulators acting *via* zinc-binding affect proteolysis of all ADAM10 substrates. All ADAM10 substrates interact with substrate secondary binding sites (exosites), however, it is conceivable that there are different sub-sets of substrates that interact with different exosites or sub-sets of exosites, which would determine a specificity of substrate-exosite interactions. Understanding which structural features of ADAM10 and its substrates determine and enable substrate-exosite interactions would then aid in the design of substrate-selective inhibitors.

**TABLE 1:** Biochemical selectivity testing of ADAMs inhibitors against a panel of zinc metalloproteinases.

**Compound**	**MMP1**	**MMP2**	**MMP8**	**MMP9**	**MMP14**	**ADAM10**	**ADAM17**
LT4^a^	346	5.4	NT	24	100	**0.04**	1.5
CID3117694^b^	>100	>100	>100	>100	>100	**1.1**	>100
GI254023X^a^	0.125	0.0021^a^	NT	0.0051	0.088	**0.027**	0.86
INCB8765^c^	>5.0	>5.0	NT	>5.0	>5.0	**0.097**	2.05
ADAM10 pro-domain^d^	NT	NT	NT	NT	NT	**0.048**	>10.0

*Synthetic substrates were used for all assays. All results are IC_50_, μM. Bold numbers indicate the potency against the main target. NT, not tested; ^*a*^from Camodeca et al. (2016); ^*b*^from Madoux et al. (2016); ^*c*^from Zhou et al. (2006); ^*d*^from Moss et al. (2007).*

### What Is Known About ADAM10 Exosites?

To date there has been only one structural study of ADAM10 ectodomain (Seegar et al., 2017) and only exosites that are described therein are in the catalytic domain. Comparison of the S1’ site of ADAM10 and ADAM17 revealed that ADAM10 S1’ site is deeper and more hydrophobic (Seegar et al., 2017), which explains the previously reported preference for bulky hydrophobic residues (Caescu et al., 2009). In a contrast, ADAM17 prefers smaller, non-aromatic hydrophobic residues (Caescu et al., 2009; Tucher et al., 2014).

Existing selective inhibitors of ADAM10 can provide additional insights into the ADAM10 secondary substrate binding sites. Differences in S1’ pocket allowed the development ADAM10 selective inhibitor LT4 (referred to as compound **3** in Camodeca et al., 2016) (ADAM10 IC_50_ = 40 nM, ADAM17 IC_50_ = 1500 nM; Camodeca et al., 2016; Zocchi et al., 2016). Molecular homology modeling using ADAM17 crystal structure as a template suggested that the hydroxamate moiety coordinates zinc of an active site, while 4-(4-cyano-2-methylphenyl) piperazinyl group interacted with residues in the S1’ tunnel.

To the best of our knowledge, there are no structural or modeling studies of interactions between ADAM10 and GI254023X, INCB8765 or ADAM10 (Moss et al., 2007). However, both GI254023X and INCB8765 have bulky aromatic groups (Figure 2) that could be interacting with S1’ exosite, which could explain their selectivity over ADAM17. Such a study of ADAM10 interactions with its pro-domain could also reveal additional previously undescribed exosites.

**FIGURE 3:**
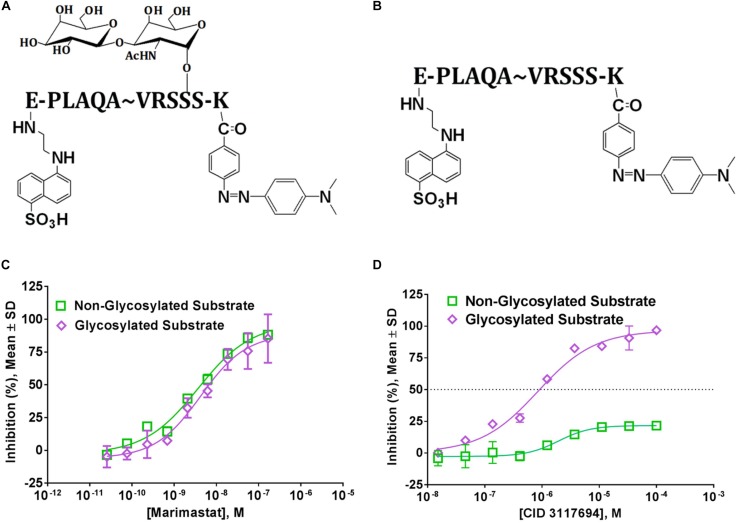
Glycosylated and non-glycosylated TNFa-based ADAM10 substrates are differentially inhibited by Zn-binding and non-Zn-binding inhibitors. Structures of **(A)** glycosylated and **(B)** non-glycosylated fluorogenic substrates. Fluorophore (Edans) and quencher (Dabcyl) are shown attached to glumatic acid (E) and lysine (K), respectively; **(C)** Proteolysis of both glycosylated and non-glycosylated substrates is inhibited equipotently by a Zn-binder marimastat, but not a non-Zn-binder CID3117694 **(D)**. Reproduced from Madoux et al. (2016) under Creative Commons License (https://creativecommons.org/licenses/by/4.0/).

LT4, GI254023X, and INCB8765 are good examples of how the targeting of ADAM10 S1’ exosite can result in metzincin-selective inhibitors. However, these ADAM10-selective compounds inhibit cleavage of all tested ADAM10 cognate substrates in the cellular models. For example, both LT4 and GI254023X prevented cleavage of activated leukocyte cell adhesion molecule (ALCAM), TNFα, MHC class I chain-related proteins A and B (MIC-A/B) and ULPBs (UL-16 binding proteins) from the surface of Hodgkin’s lymphoma cells KMH2, L428, and L540 with a very similar potency (Camodeca et al., 2016). INCB8765 did not inhibit cleavage of ADAM17-ascribed substrates (heregulin, TGFα, HB-EGF and amphiregulin), but was not tested against a panel of ADAM10-specific substrates (Zhou et al., 2006). LT4 and GI254023X testing of ADAM10 cellular substrates suggests that substrate-selective ADAM10 inhibition is difficult to achieve *via* targeting the combination of active site and S1’ exosite. As an example of targeting beyond the active site, an ADAM10 selective inhibitor, CID3117694 (Figure 2), inhibits ADAM10 *via* a non-zinc-binding competitive mechanism (Madoux et al., 2016). It exhibits a preference for inhibition of TNFα-based glycosylated substrate (Figure 3A) over its non-glycosylated variant (Figures 3B,D and Table 2) whereas a zinc-binder marimastat inhibits proteolysis of both substrates equipotently (Figure 3C). This substrate has a glycosylated Ser in the position P4’ (Minond et al., 2012) suggesting that CID3117694 competes for the exosite occupied by the disaccharide of the glycosylated substrate, presumably in the vicinity of S4’ exosite. The exact location and interacting residues in the structure of ADAM10 are not known. CID3117694 exhibits substrate selectivity as compared to GI254023X (Table 2). Most notably CID3117694 did not inhibit cleavage of HER2 and syndecan-4 when tested at 10 μM with BT474 and A549 cells, respectively, whereas GI254023X completely inhibited cleavage of both ADAM10 substrates.

**TABLE 2:** Summary of testing of ADAM10 selective inhibitors with various cell-based ADAM10 and synthetic substrates.

**Target**	**Cell line**	**Glycosylation type**	**Position**	**[C] tested, μM**	**CID3117694, %inhibition (IC_50_, μM)**	**GI254023X, %inhibition**
TNFα non-glycosylated^a^	N/A	None	N/A	0.01–100	18 (>100)	100
TNFα glycosylated^a^	N/A	Gal-GalNAc	S^4^	0.01–100	100 (1.1)	100
HER2^a^	BT474	GlcNAc	Multiple (Yuan et al., 2003)	10	0	100
CXCL16^a^	A549	Gal-GalNAc	Multiple (Abel et al., 2004)	10	80	100
Syndecan-4^a^	A549	Heparan Sulfate	S^39^, S^61^, S^63^ (Bernfield et al., 1992)	10	0	100

*NT, not tested. ^*a*^Madoux et al. (2016).*

The reason for substrate selectivity of CID3117694 is likely based on differences in glycosylation of ADAM10 substrates. CXCL16 is highly modified with mucin-like *O*-glycosylation containing galactose-*N*-acetylgalactosamine (Gal-GalNAc) as a part of its core structure within its stalk region where the cleavage by ADAM10 occurs (Abel et al., 2004). In contrast to CXCL16, syndecan-4 is *O*-glycosylated by heparan sulfate in three positions (Bernfield et al., 1992) and HER2 is *N*-glycosylated in seven positions 46–48. The substrate that was used to discover CID 3117694 is *O*-glycosylated with galactose-*N*-acetylgalactosamine (Gal-GalNAc) (Figure 1A), which suggests that CID 3117694 inhibits CXCL16 shedding by preventing its binding to the Gal-GalNAc-binding exosite in ADAM10 structure. The lack of inhibition of syndecan-4 shedding by CID 3117694 is potentially due to the fact that it cannot compete with heparan sulfate moieties which are much larger than Gal-GalNAc. Weak inhibition of HER2 shedding could be explained by the size difference between *N*-acetylglucosamine (GlcNAc, monosaccharide) found on HER2 (Franklin et al., 2004; Bostrom et al., 2009; Eigenbrot et al., 2010) and *N*-acetylgalactosamine (Gal-GalNAc, disaccharide) found on CXCL16. Another possible explanation is the distance of glycosylation site from the cleavage site. In case of the synthetic glycosylated substrate Gal-GalNAc is only four residues away from the cleavage site which is also likely the case with heavily *O*-glycosylated CXCL16, whereas in HER2 the most proximal to the cleavage site (^642^PAEQR∼ASP^650^) (Yuan et al., 2003) glycosylation N^629^ is approximately 20 residues away.

Overall, ability of CID3117694 to differentiate between ADAM10 substrates based on their glycosylation status suggests that substrate glycosylation can be used as a target for drug discovery.

### What Is Known About Glycosylation Status of ADAM10 Substrates and Its Effect on Proteolysis?

In order to be able to target an interaction between a glycan of an ADAM10 substrate and corresponding ADAM10 exosite it is necessary to know a position and type of glycan. Additionally, in order to avoid target-based toxicity, it is important that the target glycan is different in the specific diseased tissue vs. normal tissue. There are approximately hundred ADAM10 substrates (Dreymueller et al., 2015; Wetzel et al., 2017) that have been described to date, however, their glycosylation status is largely unknown. Additionally, most of information about glycosylation of ADAM10 substrates is derived from studies of healthy tissues and little is known about glycosylation of the same proteins in various diseases.

IL6 receptor (IL-6R) has four *O*-glycosylated residues nearby the ADAM cleavage site TSLPVQ^357^∼DSSSV (Table 3) that could be modulating its proteolysis (Goth et al., 2015). Additionally, an N-linked glycan on Asn^55^ of the IL-6R 302 residues away from the cleavage site, was identified as a protease regulatory exosite, whose deletion caused increased shedding of the IL-6R (Riethmueller et al., 2017). This suggests that even glycosylation far away from proteolytic site can be targeted for drug discovery. IL-6R was shown to be important in cancer (Deng et al., 2019; He et al., 2019; Weng et al., 2019; Yousefi et al., 2019) and RA (Ahmed et al., 2017) suggesting that ADAM10-mediated cleavage of IL-6R can be targeted for drug discovery for both indications. However, glycosylation profile of IL-6R in both cancer and RA is unknown.

**TABLE 3:** Results of Pubmed and UniProt database searches for information on glycosylation of ADAM10 cognate substrates available for cancer and rheumatoid arthritis.

**Accession #**	**Substrate**	**Cleavage site**	**Known glycosylation position**	**Closest distance from scissile bond, #residues**	**Glyco type in normalcy**	**Role in cancer**	**Glyco type in cancer**	**Role in RA**	**Glyco type in RA**
P35070	Pro-betacellulin	CVVA^31^/^32^DGN*S	N^34^	3	N-linked (GlcNAc) (Watanabe et al., 1994; UniProt, 2019)	Feldinger et al., 2014	Not found	Harada et al., 2015	Not found
P01375	pro-TNFa	LAQA^76^/^77^VRSS	S^80^	4	O-linked (GalNAc) (Goth et al., 2015)	Kondo et al., 1994; Janes et al., 2006; Miyazawa et al., 2008; Malekshah et al., 2012	O-linked (GalNAc) (Takakura-Yamamoto et al., 1996)	Jimi et al., 2019	Not found
P02786	Transferrin receptor	TECER^100^∼ LAGT*E	T^104^, N^251^, N^317^, N^727^	4	O-linked (GalNAc) (Do and Cummings, 1992; Hayes et al., 1992; Lawrence et al., 1999)	Shen et al., 2018	O-linked (GalNAc) (Rutledge and Enns, 1996)	Pavai et al., 2007	Not found
A0N0L5	IL6-R	T*SLPVQ^357^∼DS*S*SV	S^359^, S^360^, T^353^, N^55^, N^93^, N^221,^ N^350^	2	O-linked (GalNAc) O-linked (HexNAc) N-linked (GlcNAc) Cole et al., 1999; Goth et al., 2015	Deng et al., 2019; He et al., 2019; Weng et al., 2019; Yousefi et al., 2019	Not found	Ahmed et al., 2017	Not found
P05067	APP	*YEVHHQK^687^∼LVFFA	N^542^, N^571^, T^633^, T^651^, T^652^, S^656^, T^659^, T^663^, S^663^, S^667^, Y^681^	6	N-linked (GlcNAc) (Halim et al., 2011; Brinkmalm et al., 2012)	Wozniak and Ludwig, 2018; Wu et al., 2020	Not found	Kuroda et al., 2019	Not found
O14944	Pro-epiregulin	DNPR^59^/^60^VAQV	N^47^	12	N-linked (GlcNAc) (UniProt, 2020a)	Wang et al., 2019	Not found	Harada et al., 2015	Not found
Q99075	pro-HB-EGF	RKVR^62^/^63^DLQE	T^37^, S^38^, T^44^, T^47^, T^75^, T^85^	13	O-linked (GalNAc) (Halim et al., 2011, 2012)	Branco et al., 2019; Gelfo et al., 2019; Moore et al., 2019; Finn et al., 2020	O-linked (GalNAc) Davis-Fleische et al., 2001	Kuo et al., 2019	Not found
P01135	pro-TGFa	VAAA^39^/^40^VVSH	N^25^	14	N-linked (GlcNAc) UniProt, 2020b	Yu et al., 2018; Poteet et al., 2019	Not found	Hallbeck et al., 2005	Not found
P04626	EGFR2	AEQR^646^/^647^ASPL	N^68^, N^124^, N^187^, N^259^, N^530^, N^571^, N^629^	17	N-linked (GlcNAc) (Franklin et al., 2004; Bostrom et al., 2009; Eigenbrot et al., 2010)	Landi and Cappuzzo, 2013; Ingthorsson et al., 2016; Cirstea et al., 2017	Not found	Hallbeck et al., 2005; Shchetynsky et al., 2017	Not found
P15514	pro-Amphiregulin	IVDD^100^/^101^SVRV;	N^30^, N^113^, N^119^	18	N-linked (GlcNAc) (UniProt, 2020d)	Oliveras-Ferraros et al., 2012; Rexer et al., 2013	Not found	Nakamura et al., 2006; Yamane et al., 2008; Liu et al., 2014	Not found
P06734	CD23	EERA^61^∼RN*VSQVSKN	N^63^	2	N-linked (GlcNAc) UniProt, 2020c	Kwon et al., 2012	Not found	Rambert et al., 2009; Kuzin et al., 2016	Not found

Transferrin receptor (TfR) is shed by either ADAM10 or ADAM17 (Kaup et al., 2002). *O*-linked carbohydrate four residues away from the scissile bond (Table 3) serves to protect the TfR from proteolytic cleavage, and without this protection, the TfR is more susceptible to cleavage (Rutledge and Enns, 1996). Soluble TfR (sTfR) is used as a diagnostic test for iron deficiency anemia in rheumatoid arthritis and other diseases (Pavai et al., 2007; Berlin et al., 2011). Concentration of sTfR and, therefore, the test results, depend on glycosylation status of TfR. It is conceivable that increase of sTfR in the serum of patients could be due to the change in the glycosylation of TfR. TfR importance in cancer and RA has been demonstrated (Pavai et al., 2007; Shen et al., 2018), however, its glycosylation profile is known only for cancer (Rutledge and Enns, 1996).

Amyloid precursor protein (APP) has been studied mostly in the context of Alzheimer’s disease (AD), however, recent reports show its importance in cancer (Wozniak and Ludwig, 2018; Wu et al., 2020) and RA (Kuroda et al., 2019). While APP is glycosylated in multiple positions (Halim et al., 2011), the closest residue to the ADAM cleavage site Y^681^EVHHQK^687^∼LVFFAED is Y^681^ (Table 3). Peptides with glycosylated Y^681^ were increased in CSF of AD patients (*n* = 6) versus non-AD patients (Wu et al., 2020) suggesting that this glycosylation could be specific to AD disease state. It is not known whether APP is glycosylated at Y^681^ in cancer and RA patients.

From Table 3 it’s quite clear that substrate- and disease-specific glycosylation data necessary to target each substrate need to be obtained in order to begin a rational design or discovery of ADAM10 substrate-selective inhibitors.

### Lack of Structural Information Represents a Challenge in Using Glycosylation for Targeting

In order to be able to target a specific glyco moiety on an ADAM10 substrate there needs to be a clear understanding of what this moiety is. It would be an understatement to say that protein glycosylation is complex. It is well known that glycosylation of the same protein may differ in normalcy vs. disease [e.g., neurodegeneration (Moll et al., 2019), autoimmune disease (Li et al., 2019), type 2 diabetes, inflammatory bowel disease, or colorectal cancer (Dotz and Wuhrer, 2019)]. Additionally, glycosylation may differ based on the stage of the disease (Regan et al., 2019), age and sex of the patient (Dotz and Wuhrer, 2019), type of disease etc. Therefore, as an example, information of glycosylation of target protein available for breast cancer should not be used for diabetes. To characterize a glyco moiety present on the specific target a significant amount of a protein is required, therefore, it needs to be either expressed or isolated from disease-specific cells. Recombinant proteins are typically produced in bacteria or insect cells due to higher yield. However, because glycosylation machinery is significantly different in humans this approach is not suitable for human disease-specific analysis. This suggests that a target protein needs to be isolated and characterized for glycosylation in the specific disease scenario using either patient cells or established cell lines. This presents a challenge given that microgram to milligram quantities of protein are needed for glycomic characterization and patient cells are usually a rare commodity.

Speculatively speaking, the expression profile of glycosylating/deglycosylating enzymes could be used as a possible alternative to the glycomic characterization of target proteins. Glycosylation of ADAM10 substrates depends on the repertoire of glycosylating/deglycosylating enzymes expressed in any particular disease and tissue. As an example, an expression profile of 210 glycosyltransferase (GT) genes from 1893 cancer patients correlated well with six cancer types (Ashkani and Naidoo, 2016). Also, it correlated with clinical classification of breast cancer sub-types.

As another example, increased levels of α-2,3-sialyltransferase-1 and neuraminidase-3 in monocytes of RA patients were found to correlate with disease activity score (DAS28) (Liou and Jang, 2019) resulting in increased sialylation. It stands to reason that GT expression profile is different in various tissues and disease states, therefore, knowledge of GT expression profile could help in identifying possible glycosylation changes in the disease state. It needs to be mentioned, however, that this approach has not been experimentally tested.

### Are There Other Forms of ADAM10 Regulation Affecting Substrate Specificity?

As mentioned in Seegar et al. (2017), disintegrin/cysteine-rich domain blocks access of protein substrates to the S1’ and S2’ pockets, resulting in auto-inhibition. Binding of 8C7 F_ab_ antibody to the disintegrin/cysteine-rich domain rendered ADAM10 active suggesting that disintegrin/cysteine-rich domain might contain an exosite (or exosites) which could be used by substrates to gain access to the active site. In the original report, 8C7 F_ab_ antibody was able to inhibit ADAM10-mediated ephrin cleavage, Eph activity and Eph-dependent cell behavior (Atapattu et al., 2012). This suggests that non-catalytic domains (NCDs) participate in substrate recognition and processing and, therefore, can be targeted for drug discovery.

## Practical Considerations For Targeting Disease-Specific Glycosylation and Non-Catalytic Domains

Drug discovery targeting exosites presents unique challenges. While established methodologies can be used, need to focus on previously unexplored target class introduces a new “twist” which, in some cases, may lead to an unsurmountable technical difficulty. Here we discuss how targeting glycosylation and NCDs affects applicability of established methods of drug discovery.

### Compound Screening

Once the type and position of glycosylation of target protein is known, the researchers needs to choose an assay format for a modulator discovery. Two main approaches to drug discovery are based on either purified target (i.e., biochemical assay) or target expressed in the cells of interest (i.e., cell-based assay). Depending on a therapeutic area, activators or inhibitors of ADAM10 activity might be needed. For example, for Alzheimer’s disease the activators or potentiators of ADAM10 activity might be useful to increase non-amyloidogenic processing of APP thus decreasing amyloid plaque formation in CNS (Bandyopadhyay et al., 2007; Fahrenholz, 2007; Lichtenthaler, 2011; Postina, 2012; Manzine et al., 2019). Both biochemical and cell-based approaches have their inherent problems and advantages. Biochemical assays for ADAM10 modulators almost universally utilize synthetic fluorogenic substrates. These substrates need to be glycosylated using either chemical or chemoenzymatic approaches (Marschall et al., 2019) that are not straightforward and expensive. The synthetic substrates are significantly shorter than the native ones and typically consist of 10–15 amino acid residues. This potentially results in the lack of interactions between such a substrate with non-catalytic domains (NCDs) of ADAM10. We previously reported an effect of NCDs of ADAM10 most closely related metzincin, ADAM17, on proteolysis of TNFα-based synthetic substrates. NCDs did not directly bind the substrates used in the study but affected the binding nevertheless, most likely because of steric hindrance (Stawikowska et al., 2013). Additionally, fluorophore and quencher can interfere with binding of substrate to ADAM10. Finally, fluorogenic substrates are subject to fluorescent artifact (Marschall et al., 2019) due to intrinsically fluorescent compounds present in high-throughput screening (HTS) libraries.

Conversely, cell-based assays are more pathophysiologically relevant than biochemical assays. The target protein is present in the native form containing all possible exosites in a more complex cellular environment. Since mostly immortalized cell lines are used for HTS as a proxy for a disease model, the presence of correct glycosylation form in the right position needs to be experimentally confirmed before utilizing a particular cell line. Detection of an ADAM10 activity modulation event in cell-based assays is another potential challenge. Detection of shedding of ADAM10 target is usually dependent on an antibody-based technology (e.g., western blot, ELISA, AlphaLISA). Western blot and ELISA are not amenable to HTS leaving only AlphaLISA (or its variation, AlphaScreen) as an enabling technology for the assay development. A main consideration with using AlphaLISA is an availability of an assay kit for a specific target. If a kit for the target of interest is not commercially available, then researchers can attempt to develop their own AlphaLISA assay using commercially available antibodies that will need to be conjugated to the AlphaLISA beads. The cell-based assay using AlphaLISA will need to be developed using “addition-only” format (i.e., no supernatant transferring) meaning that ADAM10 target will need to be detected in the supernatant in the presence of live cells. In our group we were able to develop and use such an assay to discover compounds increasing soluble APPα in the supernatant of live 7WD10 cells (Wang et al., 2014) suggesting feasibility of this approach.

Overall, the choice of the approach should be based on the availability of substrate structural information and technical resources, however, it needs to be mentioned that at this stage both are sorely lacking.

### Computer-Aided Drug Design and Discovery

Another approach to target glycosylation for ADAM10 modulator discovery can be based on virtual methods such as computer modeling and/or virtual screening. Either approach requires a pre-existing knowledge of an interaction site between a ligand and a target. In the case of ADAM10, such information is not available. This suggests a need for making a working virtual model by either docking a glycosylated substrate or other known exosite ligand (e.g., CID3117694). Once such a model is available, a medicinal chemist can use interactions between ADAM10 exosite and ligand revealed as a result of modeling effort to design a small molecule. Alternatively, a virtual screening can be performed using *de novo* model and publicly available virtual compound libraries (e.g., https://zinc.docking.org) to generate hits, which will need to be confirmed in ADAM10 assay.

## Conclusion

Recent publications by different research groups independently demonstrated that glycosylation can affect ADAM10-mediated proteolysis. Research conducted in our group in the last 9 years has demonstrated that it is possible to target glycosylation of ADAM10 and ADAM17 for enzyme- and substrate-selective inhibitor discovery. This suggests that proteolysis of specific ADAM10 substrates involved in various diseases can be targeted using information about their glycosylation and non-catalytic domains differences.

## Author Contributions

DM envisioned and wrote the manuscript.

## Conflict of Interest

The authors declare that the research was conducted in the absence of any commercial or financial relationships that could be construed as a potential conflict of interest.

## References

[B1] Abel S. Hundhausen C. Mentlein R. Schulte A. Berkhout T. A. Broadway N. (2004). The transmembrane CXC-chemokine ligand 16 is induced by IFN-gamma and TNF-alpha and shed by the activity of the disintegrin-like metalloproteinase ADAM10. *J Immunol.* 172 6362–6372. 10.4049/jimmunol.172.10.6362 15128827

[B2] Ahmed S. Hussain S. Ammar A. Jahan S. Khaliq S. Kaul H. (2017). Interleukin 6 Receptor (IL6-R) Gene Polymorphisms Underlie Susceptibility to Rheumatoid Arthritis. *Clin Lab.* 63 1365–1369. 10.7754/Clin.Lab.2017.170216 28879718

[B3] Ashkani J. Naidoo K. J. (2016). Glycosyltransferase Gene Expression Profiles Classify Cancer Types and Propose Prognostic Subtypes. *Sci Rep.* 6 26451. 10.1038/srep26451 27198045 PMC4873817

[B4] Atapattu L. Saha N. Llerena C. Vail M. E. Scott A. M. Nikolov D. B. (2012). Antibodies binding the ADAM10 substrate recognition domain inhibit Eph function. *J Cell Sci.* 125(Pt 24), 6084–6093. 10.1242/jcs.112631 23108669 PMC3585520

[B5] Bandyopadhyay S. Goldstein L. E. Lahiri D. K. Rogers J. T. (2007). Role of the APP non-amyloidogenic signaling pathway and targeting alpha-secretase as an alternative drug target for treatment of Alzheimer’s disease. *Curr Med Chem.* 14 2848–2864. 10.2174/092986707782360060 18045131

[B6] Berlin T. Meyer A. Rotman-Pikielny P. Natur A. Levy Y. (2011). Soluble transferrin receptor as a diagnostic laboratory test for detection of iron deficiency anemia in acute illness of hospitalized patients. *Isr Med Assoc J.* 13 96–98. 21443035

[B7] Bernfield M. Kokenyesi R. Kato M. Hinkes M. T. Spring J. Gallo R. L. (1992). Biology of the syndecans: a family of transmembrane heparan sulfate proteoglycans. *Annu Rev Cell Biol.* 8 365–393. 10.1146/annurev.cb.08.110192.002053 1335744

[B8] Bostrom J. Yu S. F. Kan D. Appleton B. A. Lee C. V. Billeci K. (2009). Variants of the antibody herceptin that interact with HER2 and VEGF at the antigen binding site. *Science.* 323 1610–1614. 10.1126/science.1165480 19299620

[B9] Bowes J. Brown A. J. Hamon J. Jarolimek W. Sridhar A. Waldron G. (2012). Reducing safety-related drug attrition: the use of in vitro pharmacological profiling. *Nat Rev Drug Discov.* 11 909–922. 10.1038/nrd3845 23197038

[B10] Branco D. C. da Costa N. M. M. Abe C. T. S. Kataoka M. Pinheiro J. J. V. Alves Junior S. M. (2019). HIF-1alpha, NOTCH1, ADAM12, and HB-EGF are overexpressed in mucoepidermoid carcinoma. *Oral Surg Oral Med Oral Pathol Oral Radiol.* 127 e8–e17. 10.1016/j.oooo.2018.09.013 30415904

[B11] Brinkmalm G. Portelius E. Ohrfelt A. Mattsson N. Persson R. Gustavsson M. K. (2012). An online nano-LC-ESI-FTICR-MS method for comprehensive characterization of endogenous fragments from amyloid beta and amyloid precursor protein in human and cat cerebrospinal fluid. *J Mass Spectrom.* 47 591–603. 10.1002/jms.2987 22576872

[B12] Caescu C. I. Jeschke G. R. Turk B. E. (2009). Active-site determinants of substrate recognition by the metalloproteinases TACE and ADAM10. *Biochem J.* 424 79–88. 10.1042/BJ20090549 19715556 PMC2774824

[B13] Camodeca C. Cuffaro D. Nuti E. Rossello A. A. D. A. M. (2019). Metalloproteinases as Potential Drug Targets. *Curr Med Chem.* 26 2661–2689. 10.2174/0929867325666180326164104 29589526

[B14] Camodeca C. Nuti E. Tepshi L. Boero S. Tuccinardi T. Stura E. A. (2016). Discovery of a new selective inhibitor of A Disintegrin And Metalloprotease 10 (ADAM-10) able to reduce the shedding of NKG2D ligands in Hodgkin’s lymphoma cell models. *Eur J Med Chem.* 111 193–201. 10.1016/j.ejmech.2016.01.053 26871660

[B15] Cerda-Costa N. Gomis-Ruth F. X. (2014). Architecture and function of metallopeptidase catalytic domains. *Protein Sci.* 23 123–144. 10.1002/pro.2400 24596965 PMC3926739

[B16] Chalaris A. Adam N. Sina C. Rosenstiel P. Lehmann-Koch J. Schirmacher P. (2010). Critical role of the disintegrin metalloprotease ADAM17 for intestinal inflammation and regeneration in mice. *J Exp Med.* 207 1617–1624. 10.1084/jem.20092366 20603312 PMC2916135

[B17] Chavaroche A. Cudic M. Giulianotti M. Houghten R. A. Fields G. B. Minond D. (2014). Glycosylation of a disintegrin and metalloprotease 17 affects its activity and inhibition. *Anal Biochem.* 449 68–75. 10.1016/j.ab.2013.12.018 24361716 PMC4334441

[B18] Cirstea A. E. Stepan A. E. Margaritescu C. Zavoi R. E. Olimid D. A. Simionescu C. E. (2017). The immunoexpression of EGFR, HER2 and HER3 in malignant serous ovarian tumors. *Rom J Morphol Embryol* 58 1269–1273. 29556616

[B19] Cole A. R. Hall N. E. Treutlein H. R. Eddes J. S. Reid G. E. Moritz R. L. (1999). Disulfide bond structure and N-glycosylation sites of the extracellular domain of the human interleukin-6 receptor. *J Biol Chem.* 274 7207–7215. 10.1074/jbc.274.11.7207 10066782

[B20] Crawford H. C. Dempsey P. J. Brown G. Adam L. Moss M. L. (2009). ADAM10 as a therapeutic target for cancer and inflammation. *Curr Pharm Des.* 15 2288–2299. 10.2174/138161209788682442 19601831

[B21] Davis-Fleische K. M. Brigstock D. R. Besner G. E. (2001). Site-directed mutagenesis of heparin-binding EGF-like growth factor (HB-EGF): analysis of O-glycosylation sites and properties. *Growth Factors.* 19 127–143. 10.3109/08977190109001081 11769972

[B22] Dekkers P. E. Lauw F. N. ten Hove T. te Velde A. A. Lumley P. Becherer D. (1999). The effect of a metalloproteinase inhibitor (GI5402) on tumor necrosis factor-alpha (TNF-alpha) and TNF-alpha receptors during human endotoxemia. *Blood.* 94 2252–2258. 10.1182/blood.v94.7.2252.419k25_2252_2258 10498596

[B23] Deng S. Wang A. Chen X. Du Q. Wu Y. Chen G. (2019). HBD Inhibits the Development of Colitis-Associated Cancer in Mice via the IL-6R/STAT3 Signaling Pathway. *Int J Mol Sci* 20 1069. 10.3390/ijms20051069 30832202 PMC6429321

[B24] Do S. I. Cummings R. D. (1992). Presence of O-linked oligosaccharide on a threonine residue in the human transferrin receptor. *Glycobiology.* 2 345–353. 10.1093/glycob/2.4.345 1421756

[B25] Dotz V. Wuhrer M. (2019). N-glycome signatures in human plasma: associations with physiology and major diseases. *FEBS Lett.* 593 2966–2976. 10.1002/1873-3468.13598 31509238

[B26] Dreymueller D. Uhlig S. Ludwig A. (2015). ADAM-family metalloproteinases in lung inflammation: potential therapeutic targets. *Am J Physiol Lung Cell Mol Physiol.* 308 L325–L343. 10.1152/ajplung.00294.2014 25480335

[B27] Eigenbrot C. Ultsch M. Dubnovitsky A. Abrahmsen L. Hard T. (2010). Structural basis for high-affinity HER2 receptor binding by an engineered protein. *Proc Natl Acad Sci U S A.* 107 15039–15044. 10.1073/pnas.1005025107 20696930 PMC2930565

[B28] Fahrenholz F. (2007). Alpha-secretase as a therapeutic target. *Curr Alzheimer Res.* 4 412–417. 10.2174/156720507781788837 17908044

[B29] Feldinger K. Generali D. Kramer-Marek G. Gijsen M. Ng T. B. Wong J. H. (2014). ADAM10 mediates trastuzumab resistance and is correlated with survival in HER2 positive breast cancer. *Oncotarget.* 5 6633–6646. 10.18632/oncotarget.1955 24952873 PMC4196152

[B30] Finn K. J. Martin S. E. Settleman J. (2020). A Single-Step, High-Dose Selection Scheme Reveals Distinct Mechanisms of Acquired Resistance to Oncogenic Kinase Inhibition in Cancer Cells. *Cancer Res.* 80 79–90. 10.1158/0008-5472.CAN-19-0729 31641034

[B31] Franklin M. C. Carey K. D. Vajdos F. F. Leahy D. J. de Vos A. M. Sliwkowski M. X. (2004). Insights into ErbB signaling from the structure of the ErbB2-pertuzumab complex. *Cancer Cell.* 5 317–328. 10.1016/s1535-6108(04)00083-2 15093539

[B32] Gelfo V. Pontis F. Mazzeschi M. Sgarzi M. Mazzarini M. Solmi R. (2019). Glucocorticoid Receptor Modulates EGFR Feedback upon Acquisition of Resistance to Monoclonal Antibodies. *J Clin Med* 8 600. 10.3390/jcm8050600 31052457 PMC6572202

[B33] Goth C. K. Halim A. Khetarpal S. A. Rader D. J. Clausen H. Schjoldager K. T. (2015). A systematic study of modulation of ADAM-mediated ectodomain shedding by site-specific O-glycosylation. *Proc Natl Acad Sci U S A.* 112 14623–14628. 10.1073/pnas.1511175112 26554003 PMC4664366

[B34] Halim A. Brinkmalm G. Ruetschi U. Westman-Brinkmalm A. Portelius E. Zetterberg H. (2011). Site-specific characterization of threonine, serine, and tyrosine glycosylations of amyloid precursor protein/amyloid beta-peptides in human cerebrospinal fluid. *Proc Natl Acad Sci U S A.* 108 11848–11853. 10.1073/pnas.1102664108 21712440 PMC3141957

[B35] Halim A. Nilsson J. Ruetschi U. Hesse C. Larson G. (2012). Human urinary glycoproteomics; attachment site specific analysis of N- and O-linked glycosylations by CID and ECD. *Mol Cell Proteomics.* 11 M111013649. 10.1074/mcp.M111.013649 22171320 PMC3322569

[B36] Hallbeck A. L. Walz T. M. Briheim K. Wasteson A. (2005). TGF-alpha and ErbB2 production in synovial joint tissue: increased expression in arthritic joints. *Scand J Rheumatol* 34 204–211. 10.1080/03009740510017715 16134726

[B37] Harada M. Kamimura D. Arima Y. Kohsaka H. Nakatsuji Y. Nishida M. (2015). Temporal expression of growth factors triggered by epiregulin regulates inflammation development. *J Immunol.* 194 1039–1046. 10.4049/jimmunol.1400562 25556244

[B38] Hartmann D. de Strooper B. Serneels L. Craessaerts K. Herreman A. Annaert W. (2002). The disintegrin/metalloprotease ADAM 10 is essential for Notch signalling but not for alpha-secretase activity in fibroblasts. *Hum Mol Genet.* 11 2615–2624. 10.1093/hmg/11.21.2615 12354787

[B39] Hayes G. R. Enns C. A. Lucas J. J. (1992). Identification of the O-linked glycosylation site of the human transferrin receptor. *Glycobiology.* 2 355–359. 10.1093/glycob/2.4.355 1421757

[B40] He B. Pan B. Pan Y. Sun H. Xu T. Qin J. (2019). IL-4/IL-4R and IL-6/IL-6R genetic variations and gastric cancer risk in the Chinese population. *Am J Transl Res.* 11 3698–3706. 31312381 PMC6614618

[B41] Ingthorsson S. Andersen K. Hilmarsdottir B. Maelandsmo G. M. Magnusson M. K. Gudjonsson T. (2016). HER2 induced EMT and tumorigenicity in breast epithelial progenitor cells is inhibited by coexpression of EGFR. *Oncogene* 35 4244–4255. 10.1038/onc.2015.489 26686087 PMC4981873

[B42] Janes K. A. Gaudet S. Albeck J. G. Nielsen U. B. Lauffenburger D. A. Sorger P. K. (2006). The response of human epithelial cells to TNF involves an inducible autocrine cascade. *Cell.* 124 1225–1239. 10.1016/j.cell.2006.01.041 16564013

[B43] Jimi E. Fei H. Nakatomi C. (2019). NF-kappaB Signaling Regulates Physiological and Pathological Chondrogenesis. *Int J Mol Sci* 20 6275. 10.3390/ijms20246275 31842396 PMC6941088

[B44] Kaup M. Dassler K. Weise C. Fuchs H. (2002). Shedding of the transferrin receptor is mediated constitutively by an integral membrane metalloprotease sensitive to tumor necrosis factor alpha protease inhibitor-2. *J Biol Chem.* 277 38494–38502. 10.1074/jbc.M203461200 12163483

[B45] Kondo S. Yin D. Takeuchi J. Morimura T. Miyatake S. I. Nakatsu S. (1994). Tumour necrosis factor-alpha induces an increase in susceptibility of human glioblastoma U87-MG cells to natural killer cell-mediated lysis. *British journal of cancer.* 69 627–632. 10.1038/bjc.1994.123 7908214 PMC1968817

[B46] Koo C. Z. Harrison N. Noy P. J. Szyroka J. Matthews A. L. Hsia H. E. (2020). The tetraspanin Tspan15 is an essential subunit of an ADAM10 scissor complex. *J Biol Chem* 10.1074/jbc.RA120.012601 32111735 PMC7476718

[B47] Kuhn P. H. Colombo A. V. Schusser B. Dreymueller D. Wetzel S. Schepers U. (2016). Systematic substrate identification indicates a central role for the metalloprotease ADAM10 in axon targeting and synapse function. *Elife* 5 e012748. 10.7554/eLife.12748 26802628 PMC4786429

[B48] Kuo D. Ding J. Cohn I. S. Zhang F. Wei K. Rao D. A. (2019). HBEGF(+) macrophages in rheumatoid arthritis induce fibroblast invasiveness. *Sci Transl Med* 11 eaau8587. 10.1126/scitranslmed.aau8587 31068444 PMC6726376

[B49] Kuroda T. Ito Y. Imai N. Nozawa Y. Sato H. Nakatsue T. (2019). Significant association between renal function and area of amyloid deposition in kidney biopsy specimens from patients with AA amyloidosis associated with rheumatoid arthritis and AL amyloidosis. *Amyloid* 26 125–126. 10.1080/13506129.2019.1582512 31343309

[B50] Kuzin I. I. Kates S. L. Ju Y. Zhang L. Rahimi H. Wojciechowski W. (2016). Increased numbers of CD23(+) CD21(hi) Bin-like B cells in human reactive and rheumatoid arthritis lymph nodes. *Eur J Immunol.* 46 1752–1757. 10.1002/eji.201546266 27105894 PMC4942352

[B51] Kwon H. S. Park M. C. Kim D. G. Cho K. Park Y. W. Han J. M. (2012). Identification of CD23 as a functional receptor for the proinflammatory cytokine AIMP1/p43. *J Cell Sci.* 125(Pt 19), 4620–4629. 10.1242/jcs.108209 22767513

[B52] Landi L. Cappuzzo F. (2013). HER2 and lung cancer. *Expert Rev Anticancer Ther* 13 1219–1228. 10.1586/14737140.2013.846830 24134423

[B53] Lawrence C. M. Ray S. Babyonyshev M. Galluser R. Borhani D. W. Harrison S. C. (1999). Crystal structure of the ectodomain of human transferrin receptor. *Science.* 286 779–782. 10.1126/science.286.5440.779 10531064

[B54] Li X. Xu J. Li M. Zeng X. Wang J. Hu C. (2019). Aberrant glycosylation in autoimmune disease. *Clin Exp Rheumatol^∗^* 31694739

[B55] Lichtenthaler S. F. (2011). Alpha-secretase in Alzheimer’s disease: molecular identity, regulation and therapeutic potential. *J Neurochem.* 116 10–21. 10.1111/j.1471-4159.2010.07081.x 21044078

[B56] Liou L. B. Jang S. S. (2019). Alpha-2,3-Sialyltransferase 1 and neuraminidase-3 from monocytes in patients with rheumatoid arthritis correlate with disease activity measures: A pilot study. *J Chin Med Assoc.* 82 179–185. 10.1097/JCMA.0000000000000027 30913115

[B57] Liu F. L. Wu C. C. Chang D. M. (2014). TACE-dependent amphiregulin release is induced by IL-1beta and promotes cell invasion in fibroblast-like synoviocytes in rheumatoid arthritis. *Rheumatology (Oxford)* 53 260–269. 10.1093/rheumatology/ket350 24196392

[B58] Lorenzen I. Lokau J. Korpys Y. Oldefest M. Flynn C. M. Kunzel U. (2016). Control of ADAM17 activity by regulation of its cellular localisation. *Sci Rep.* 6 35067. 10.1038/srep35067 27731361 PMC5059621

[B59] Ludwig A. Hundhausen C. Lambert M. H. Broadway N. Andrews R. C. Bickett D. M. (2005). Metalloproteinase inhibitors for the disintegrin-like metalloproteinases ADAM10 and ADAM17 that differentially block constitutive and phorbol ester-inducible shedding of cell surface molecules. *Comb Chem High Throughput Screen.* 8 161–171. 10.2174/1386207053258488 15777180

[B60] Madoux F. Dreymuller D. Pettiloud J. P. Santos R. Becker-Pauly C. Ludwig A. (2016). Discovery of an enzyme and substrate selective inhibitor of ADAM10 using an exosite-binding glycosylated substrate. *Sci Rep.* 6 11. 10.1038/s41598-016-0013-4 28442704 PMC5431342

[B61] Malekshah O. M. Lage H. Bahrami A. R. Afshari J. T. Behravan J. (2012). PXR and NF-kappaB correlate with the inducing effects of IL-1beta and TNF-alpha on ABCG2 expression in breast cancer cell lines. *Eur J Pharm Sci.* 47 474–480. 10.1016/j.ejps.2012.06.011 22750628

[B62] Manzine P. R. Ettcheto M. Cano A. Busquets O. Marcello E. Pelucchi S. (2019). ADAM10 in Alzheimer’s disease: Pharmacological modulation by natural compounds and its role as a peripheral marker. *Biomed Pharmacother.* 113 108661. 10.1016/j.biopha.2019.108661 30836275

[B63] Marschall E. Cryle M. J. Tailhades J. (2019). Biological, chemical, and biochemical strategies for modifying glycopeptide antibiotics. *J Biol Chem.* 294 18769–18783. 10.1074/jbc.REV119.006349 31672921 PMC6901329

[B64] Matthews A. L. Noy P. J. Reyat J. S. Tomlinson M. G. (2017). Regulation of A disintegrin and metalloproteinase (ADAM) family sheddases ADAM10 and ADAM17: The emerging role of tetraspanins and rhomboids. *Platelets.* 28 333–341. 10.1080/09537104.2016.1184751 27256961 PMC5490636

[B65] Minond D. Cudic M. Bionda N. Giulianotti M. Maida L. Houghten R. A. (2012). Discovery of novel inhibitors of a disintegrin and metalloprotease 17 (ADAM17) using glycosylated and non-glycosylated substrates. *J Biol Chem.* 287 36473–36487. 10.1074/jbc.M112.389114 22927435 PMC3476313

[B66] Miyazawa M. Ito Y. Kosaka N. Nukada Y. Sakaguchi H. Suzuki H. (2008). Role of TNF-alpha and extracellular ATP in THP-1 cell activation following allergen exposure. *J Toxicol Sci.* 33 71–83. 10.2131/jts.33.71 18303186

[B67] Moll T. Shaw P. J. Cooper-Knock J. (2019). Disrupted glycosylation of lipids and proteins is a cause of neurodegeneration. *Brain*^∗^ 10.1093/brain/awz358 31724708 PMC7241952

[B68] Moore K. N. Bendell J. C. LoRusso P. M. Olszanski A. J. Zwick-Wallasch E. Jansen M. (2019). First-in-human study of the anti-HB-EGF antibody U3-1565 in subjects with advanced solid tumors. *Invest New Drugs.* 37 147–158. 10.1007/s10637-018-0646-1 30056611 PMC6886232

[B69] Moss M. L. Bartsch J. W. (2004). Therapeutic benefits from targeting of ADAM family members. *Biochemistry.* 43 7227–7235. 10.1021/bi049677f 15182168

[B70] Moss M. L. Minond D. (2017). Recent Advances in ADAM17 Research: A Promising Target for Cancer and Inflammation. *Mediators Inflamm.* 2017 9673537. 10.1155/2017/9673537 29230082 PMC5688260

[B71] Moss M. L. Bomar M. Liu Q. Sage H. Dempsey P. Lenhart P. M. (2007). The ADAM10 prodomain is a specific inhibitor of ADAM10 proteolytic activity and inhibits cellular shedding events. *J Biol Chem.* 282 35712–35721. 10.1074/jbc.M703231200 17895248

[B72] Moss M. L. Sklair-Tavron L. Nudelman R. (2008a). Drug insight: tumor necrosis factor-converting enzyme as a pharmaceutical target for rheumatoid arthritis. *Nat Clin Pract Rheumatol.* 4 300–309. 10.1038/ncprheum0797 18414459

[B73] Moss M. L. Stoeck A. Yan W. Dempsey P. J. (2008b). ADAM10 as a target for anti-cancer therapy. *Curr Pharm Biotechnol.* 9 2–8. 10.2174/138920108783497613 18289051

[B74] Nakamura N. Shimaoka Y. Tougan T. Onda H. Okuzaki D. Zhao H. (2006). Isolation and expression profiling of genes upregulated in bone marrow-derived mononuclear cells of rheumatoid arthritis patients. *DNA Res.* 13 169–183. 10.1093/dnares/dsl006 17082220

[B75] Newton R. C. Solomon K. A. Covington M. B. Decicco C. P. Haley P. J. Friedman S. M. (2001). Biology of TACE inhibition. *Annals of the rheumatic diseases.* 60 (Suppl. 3), iii25–iii32. 11890648 10.1136/ard.60.90003.iii25 PMC1766675

[B76] Oliveras-Ferraros C. Cufi S. Queralt B. Vazquez-Martin A. Martin-Castillo B. de Llorens R. (2012). Cross-suppression of EGFR ligands amphiregulin and epiregulin and de-repression of FGFR3 signalling contribute to cetuximab resistance in wild-type KRAS tumour cells. *British journal of cancer.* 106 1406–1414. 10.1038/bjc.2012.103 22491422 PMC3326676

[B77] Overall C. M. Lopez-Otin C. (2002). Strategies for MMP inhibition in cancer: innovations for the post-trial era. *Nat Rev Cancer.* 2 657–672. 10.1038/nrc884 12209155

[B78] Pavai S. Jayaranee S. Sargunan S. (2007). Soluble transferrin receptor, ferritin and soluble transferrin receptor–Ferritin index in assessment of anaemia in rhaeumatoid arthritis. *Med J Malaysia.* 62 303–307. Epub 2008/06/17., 18551934

[B79] Postina R. (2012). Activation of alpha-secretase cleavage. *J Neurochem.* 120 (Suppl. 1), 46–54. 10.1111/j.1471-4159.2011.07459.x 21883223

[B80] Poteet E. Liu D. Liang Z. Van Buren G. Chen C. Yao Q. (2019). Mesothelin and TGF-alpha predict pancreatic cancer cell sensitivity to EGFR inhibitors and effective combination treatment with trametinib. *PLoS One.* 14:e0213294. 10.1371/journal.pone.0213294 30921351 PMC6438513

[B81] Pruessmeyer J. Ludwig A. (2009). The good, the bad and the ugly substrates for ADAM10 and ADAM17 in brain pathology, inflammation and cancer. *Semin Cell Dev Biol.* 20 164–174. 10.1016/j.semcdb.2008.09.005 18951988

[B82] Rambert J. Mamani-Matsuda M. Moynet D. Dubus P. Desplat V. Kauss T. (2009). Molecular blocking of CD23 supports its role in the pathogenesis of arthritis. *PLoS One* 4:e4834. 10.1371/journal.pone.0004834 19279679 PMC2652713

[B83] Regan P. McClean P. L. Smyth T. Doherty M. (2019). Early Stage Glycosylation Biomarkers in Alzheimer’s Disease. *Medicines (Basel).* 6 92. 10.3390/medicines6030092 31484367 PMC6789538

[B84] Reiss K. Bhakdi S. (2017). The plasma membrane: Penultimate regulator of ADAM sheddase function. *Biochim Biophys Acta Mol Cell Res.* 1864(11 Pt B), 2082–2087. 10.1016/j.bbamcr.2017.06.006 28624437

[B85] Rexer B. N. Ghosh R. Narasanna A. Estrada M. V. Chakrabarty A. Song Y. (2013). Human breast cancer cells harboring a gatekeeper T798M mutation in HER2 overexpress EGFR ligands and are sensitive to dual inhibition of EGFR and HER2. *Clin Cancer Res* 19 5390–5401. 10.1158/1078-0432.ccr-13-1038 23948973 PMC3809918

[B86] Riethmueller S. Somasundaram P. Ehlers J. C. Hung C. W. Flynn C. M. Lokau J. (2017). Proteolytic Origin of the Soluble Human IL-6R In Vivo and a Decisive Role of N-Glycosylation. *PLoS Biol.* 15:e2000080. 10.1371/journal.pbio.2000080 28060820 PMC5218472

[B87] Rutledge E. A. Enns C. A. (1996). Cleavage of the Transferrin Receptor Is Influenced by the Composition of the 0-Linked Carbohydrate at Position 104. *Journal Of Cellular Physiology* 168 284–293. 10.1002/(sici)1097-4652(199608)168:2<284::aid-jcp7>3.0.co;2-l 8707864

[B88] Saftig P. Lichtenthaler S. F. (2015). The alpha secretase ADAM10: A metalloprotease with multiple functions in the brain. *Prog Neurobiol.* 135 1–20. 10.1016/j.pneurobio.2015.10.003 26522965

[B89] Saha N. Robev D. Himanen J. P. Nikolov D. B. (2019). ADAM proteases: Emerging role and targeting of the non-catalytic domains. *Cancer Lett.* 467 50–57. 10.1016/j.canlet.2019.10.003 31593799 PMC7485987

[B90] Scharfenberg F. Helbig A. Sammel M. Benzel J. Schlomann U. Peters F. (2019). Degradome of soluble ADAM10 and ADAM17 metalloproteases. *Cell Mol Life Sci.* 77 331–350. 10.1007/s00018-019-03184-4 31209506 PMC11105009

[B91] Seegar T. C. M. Killingsworth L. B. Saha N. Meyer P. A. Patra D. Zimmerman B. (2017). Structural Basis for Regulated Proteolysis by the alpha-Secretase ADAM10. *Cell* 171 1638–48e7. 10.1016/j.cell.2017.11.014 29224781 PMC5773094

[B92] Seipold L. Altmeppen H. Koudelka T. Tholey A. Kasparek P. Sedlacek R. (2018). In vivo regulation of the A disintegrin and metalloproteinase 10 (ADAM10) by the tetraspanin 15. *Cell Mol Life Sci.* 75 3251–3267. 10.1007/s00018-018-2791-2 29520422 PMC11105247

[B93] Shchetynsky K. Diaz-Gallo L. M. Folkersen L. Hensvold A. H. Catrina A. I. Berg L. (2017). Discovery of new candidate genes for rheumatoid arthritis through integration of genetic association data with expression pathway analysis. *Arthritis Res Ther* 19 19. 10.1186/s13075-017-1220-5 28148290 PMC5288892

[B94] Shen Y. Li X. Dong D. Zhang B. Xue Y. Shang P. (2018). Transferrin receptor 1 in cancer: a new sight for cancer therapy. *Am J Cancer Res.* 8 916–931. 30034931 PMC6048407

[B95] Stawikowska R. Cudic M. Giulianotti M. Houghten R. A. Fields G. B. Minond D. (2013). Activity of ADAM17 (a disintegrin and metalloprotease 17) is regulated by its noncatalytic domains and secondary structure of its substrates. *J Biol Chem.* 288 22871–22879. 10.1074/jbc.M113.462267 23779109 PMC3829370

[B96] Takakura-Yamamoto R. Yamamoto S. Fukuda S. Kurimoto M. (1996). O-glycosylated species of natural human tumor-necrosis factor-alpha. *Eur J Biochem.* 235 431–437. 10.1111/j.1432-1033.1996.00431.x 8631363

[B97] Takeda S. (2009). Three-dimensional domain architecture of the ADAM family proteinases. *Semin Cell Dev Biol.* 20 146–152. 10.1016/j.semcdb.2008.07.009 18706512

[B98] Takeda S. (2016). ADAM and ADAMTS Family Proteins and Snake Venom Metalloproteinases: A Structural Overview. *Toxins.* 8 155. 10.3390/toxins8050155 27196928 PMC4885070

[B99] Tape C. J. Willems S. H. Dombernowsky S. L. Stanley P. L. Fogarasi M. Ouwehand W. (2011). Cross-domain inhibition of TACE ectodomain. *Proc Natl Acad Sci U S A.* 108 5578–5583. 10.1073/pnas.1017067108 21415364 PMC3078358

[B100] Tucher J. Linke D. Koudelka T. Cassidy L. Tredup C. Wichert R. (2014). LC-MS based cleavage site profiling of the proteases ADAM10 and ADAM17 using proteome-derived peptide libraries. *Journal of proteome research.* 13 2205–2214. 10.1021/pr401135u 24635658

[B101] UniProt (2020c). *UniProtKB - P15514 (AREG_HUMAN).* Available from: https://www.uniprot.org/uniprot/P15514#ptm_processing

[B102] UniProt (2019). *UniProtKB - P35070 (BTC_HUMAN).* Available from: https://www.uniprot.org/uniprot/P35070

[B103] UniProt (2020a). *UniProtKB - O14944 (EREG_HUMAN)*. Available online at: https://www.uniprot.org/uniprot/O14944#ptm_processing

[B104] UniProt (2020b). *UniProtKB - P01135 (TGFA_HUMAN).* Available from: https://www.uniprot.org/uniprot/P01135

[B105] UniProt (2020d). *UniProtKB - P06734 (FCER2_HUMAN).* Available online at: https://www.uniprot.org/uniprot/P06734#ptm_processing 10.1093/dnares/dsl006

[B106] Wang H. Nefzi A. Fields G. B. Lakshmana M. K. Minond D. (2014). AlphaLISA-based high-throughput screening assay to measure levels of soluble amyloid precursor protein alpha. *Anal Biochem.* 459 24–30. 10.1016/j.ab.2014.05.007 24857774

[B107] Wang Y. Jing Y. Ding L. Zhang X. Song Y. Chen S. (2019). Epiregulin reprograms cancer-associated fibroblasts and facilitates oral squamous cell carcinoma invasion via JAK2-STAT3 pathway. *J Exp Clin Cancer Res.* 38 274. 10.1186/s13046-019-1277-x 31234944 PMC6591968

[B108] Watanabe T. Shintani A. Nakata M. Shing Y. Folkman J. Igarashi K. (1994). Recombinant human betacellulin. Molecular structure, biological activities, and receptor interaction. *J Biol Chem.* 269 9966–9973. 8144591

[B109] Weng Y. S. Tseng H. Y. Chen Y. A. Shen P. C. Al Haq A. T. Chen L. M. (2019). MCT-1/miR-34a/IL-6/IL-6R signaling axis promotes EMT progression, cancer stemness and M2 macrophage polarization in triple-negative breast cancer. *Mol Cancer.* 18 42. 10.1186/s12943-019-0988-0 30885232 PMC6421700

[B110] Wetzel S. Seipold L. Saftig P. (2017). The metalloproteinase ADAM10: A useful therapeutic target? *Biochim Biophys Acta Mol Cell Res.* 1864(11 Pt B), 2071–2081. 10.1016/j.bbamcr.2017.06.005 28624438

[B111] Willems S. H. Tape C. J. Stanley P. L. Taylor N. A. Mills I. G. Neal D. E. (2010). Thiol isomerases negatively regulate the cellular shedding activity of ADAM17. *Biochem J.* 428 439–450. 10.1042/BJ20100179 20345372

[B112] Wozniak J. Ludwig A. (2018). Novel role of APP cleavage by ADAM10 for breast cancer metastasis. *EBioMedicine.* 38 5–6. 10.1016/j.ebiom.2018.11.050 30503863 PMC6306391

[B113] Wu X. Chen S. Lu C. (2020). Amyloid precursor protein promotes the migration and invasion of breast cancer cells by regulating the MAPK signaling pathway. *Int J Mol Med.* 45 162–174. 10.3892/ijmm.2019.4404 31746365 PMC6889931

[B114] Yamane S. Ishida S. Hanamoto Y. Kumagai K. Masuda R. Tanaka K., et al. (2008). Proinflammatory role of amphiregulin, an epidermal growth factor family member whose expression is augmented in rheumatoid arthritis patients. *J Inflamm* 5 5. 10.1186/1476-9255-5-5 18439312 PMC2396620

[B115] Yiotakis A. Dive V. (2008). Synthetic active site-directed inhibitors of metzincins: achievement and perspectives. *Mol Aspects Med.* 29 329–338. 10.1016/j.mam.2008.06.001 18657570

[B116] Yousefi H. Momeny M. Ghaffari S. H. Parsanejad N. Poursheikhani A. Javadikooshesh S. (2019). IL-6/IL-6R pathway is a therapeutic target in chemoresistant ovarian cancer. *Tumori.* 105 84–91. 10.1177/0300891618784790 30021477

[B117] Yu C. Y. Chang W. C. Zheng J. H. Hung W. H. Cho E. C. (2018). Transforming growth factor alpha promotes tumorigenesis and regulates epithelial-mesenchymal transition modulation in colon cancer. *Biochem Biophys Res Commun.* 506 901–906. 10.1016/j.bbrc.2018.10.137 30392905

[B118] Yuan C. X. Lasut A. L. Wynn R. Neff N. T. Hollis G. F. Ramaker M. L. (2003). Purification of Her-2 extracellular domain and identification of its cleavage site. *Protein Expr Purif.* 29 217–222. 10.1016/s1046-5928(03)00058-5 12767812

[B119] Zhou B. B. Peyton M. He B. Liu C. Girard L. Caudler E. (2006). Targeting ADAM-mediated ligand cleavage to inhibit HER3 and EGFR pathways in non-small cell lung cancer. *Cancer Cell.* 10 39–50. 10.1016/j.ccr.2006.05.024 16843264 PMC4451119

[B120] Zocchi M. R. Camodeca C. Nuti E. Rossello A. Vene R. Tosetti F. (2016). ADAM10 new selective inhibitors reduce NKG2D ligand release sensitizing Hodgkin lymphoma cells to NKG2D-mediated killing. *Oncoimmunology.* 5 e1123367. 10.1080/2162402X.2015.1123367 27467923 PMC4910733

